# Effects of Topical Anti-Glaucoma Medications on Outcomes of Endoscopic Dacryocystorhinostomy: Comparison with Age- and Sex-Matched Controls

**DOI:** 10.3390/jcm13020634

**Published:** 2024-01-22

**Authors:** Seong Eun Lee, Hyung Bin Lim, Seungjun Oh, Kibum Lee, Sung Bok Lee

**Affiliations:** 1 Department of Ophthalmology, Chungbuk National University Hospital, College of Medicine, Chungbuk National University, Cheongju 28644, Republic of Korea; selee@cbnuh.or.kr (S.E.L.); kibum420@cbnuh.or.kr (K.L.); 2 Department of Ophthalmology, College of Medicine, Chungbuk National University, Daejeon 35015, Republic of Korea; 20210141@cnuh.co.kr (S.O.); 3 1.0 Eye Clinic, Daejeon 34946, Republic of Korea; cromfans@hanmail.net (H.B.L.)

**Keywords:** topical anti-glaucoma medication, endoscopic dacryocystorhinostomy, nasolacrimal duct obstruction, surgical outcome

## Abstract

Background: This study analyzed the effects of topical anti-glaucoma medications on the surgical outcomes of endoscopic dacryocystorhinostomy (EDCR) in nasolacrimal duct obstruction (NLDO). Methods: This retrospective study included patients who underwent EDCR for NLDO between September 2012 and April 2021. Thirty patients with topical anti-glaucoma medications and 90 age- and sex-matched controls were included. Results: The success rate of EDCR was higher in the control group than in the anti-glaucoma group (97.8% vs. 86.7%, *p* = 0.034). Univariate and multivariate logistic regression analyses identified prostaglandin analogs as the most influential risk factor for EDCR success among anti-glaucoma medication ingredients (*p* = 0.005). The success rate of the group containing all four anti-glaucoma medication ingredients was statistically significant (*p* = 0.010). The success rate was significantly different in the group of patients who used anti-glaucoma medication for >24 months (*p* = 0.019). When multiplying the number of drug ingredients by the duration in months, the group > 69 showed a significantly decreased success rate (*p* = 0.022). Multivariate logistic regression analysis identified the number of anti-glaucoma medications as the most significant risk factor for EDCR success (odds ratio, 0.437; 95% confidence interval, 0.247 to 0.772; *p* = 0.004). Conclusions: The authors suggest that the anti-glaucoma medications might cause NLDO and increase the failure rate after EDCR. Therefore, when performing EDCR in patients using topical anti-glaucoma medications, surgeons should consider the possibility of increased recurrence after EDCR in clinical outcomes.

## 1. Introduction

The etiology of nasolacrimal duct obstruction (NLDO) has not been completely elucidated; however, idiopathic inflammation and fibrosis are known to lead to nasolacrimal duct (NLD) stenosis [1]. According to previous studies, 5–23% [2,3,4] of patients are pre-diagnosed with glaucoma when they are diagnosed with lacrimal drainage system obstruction. Anti-glaucoma medications may induce ocular surface diseases (OSD) such as conjunctival inflammation and subconjunctival fibrosis in patients [5,6]. The prevalence of OSD in patients with glaucoma due to the use of anti-glaucoma medications has been reported to be 17–52.3% in Asian populations and 40–60% in Western populations [5,7]. Active ingredients, preservatives, and excipients of anti-glaucoma medications are thought to cause ocular surface toxicity [3,7,8]. Several studies have inferred that anti-glaucoma medications may induce inflammation and fibrosis in the NLD mucosa, like how they affect the ocular surface, potentially leading to NLDO development [3,9].

Topical anti-glaucoma medications may also have a similar mechanism, affecting the newly created drainage pathway after dacryocystorhinostomy (DCR) and potentially influencing surgical outcomes. Therefore, when performing DCR on patients with concomitant glaucoma who have been using anti-glaucoma medications, it is important to consider the potential impact on surgical outcomes. Di Maria et al. [9] inferred that anti-glaucoma medications may damage the spiral fibers of the mucous membrane of the lacrimal system, induce fibrosis via a pro-inflammatory mechanism, and subsequently lead to decreased propulsive ability after DCR. This study aimed to compare the surgical success rates of patients using anti-glaucoma medications with those of an age- and sex-matched control group to investigate the impact of anti-glaucoma medications on DCR.

## 2. Materials and Methods

A retrospective review of the medical records of 795 patients who underwent endoscopic dacryocystorhinostomy (EDCR) procedures for NLDO at Chungnam National University Hospital between September 2012 and April 2021 was conducted. Patients who did not exhibit severe alterations in the osteomeatal complex, which could potentially affect the success rate of EDCR, were enrolled based on orbital computed tomography (CT) scans to standardize the surgical procedure. Severe scar formation or synechia resulting from trauma, tumors, or previous operations, potentially contributing to the failure of DCR, were excluded. During this period, 30 patients (3.8%) had a history of receiving topical anti-glaucoma medications. We included an age- and sex-matched control group of 90 patients.

Patients with glaucoma underwent additional ophthalmic examinations, including Goldmann applanation tonometry, fundus photography (TRC-NW8 fundus camera; Topcon Medical Systems, Tokyo, Japan), SD-OCT (Cirrus HD OCT; Carl Zeiss Meditec, Dublin, CA, USA), and the 24-2 Swedish Interactive Threshold Algorithm standard automated visual field test (Humphrey Visual Field Analyzer; Carl Zeiss Meditec, Dublin, CA, USA). Glaucoma was diagnosed based on glaucomatous optic disc changes and a reproducible glaucomatous visual field (VF) defect on Humphrey perimetry. Glaucomatous optic disc changes were defined as follows: diffuse or focal rim thinning, cupping, or retinal nerve fiber layer defects with corresponding VF defects. Glaucomatous VF defects were defined if they met two of the following three criteria: the presence of a cluster of three points on a pattern deviation probability plot at *p* < 0.05, one of which was at *p* < 0.01, a pattern standard deviation at *p* < 0.05, or glaucoma hemifield test results outside normal limits [10,11,12].

Anatomical success was defined as the observation of water passage during lacrimal irrigation with an open ostium confirmed through nasal endoscopy. The minimum required follow-up period was 6 months after surgery. The exclusion criteria were as follows: uncertain anti-glaucoma medication history; change in anti-glaucoma medications 6 months before EDCR surgery; less than 6 months of follow-up after EDCR surgery; patients who developed increased intraocular pressure as steroid responders after EDCR; history of congenital obstruction; eyelid margin malposition; previous lacrimal drainage system surgery; ocular or periocular trauma; history of systemic chemotherapy; and orbital radiotherapy. The study protocol was approved by the Institutional Review Board of the Chungnam National University Hospital (IRB no. 2023-03-016) and adhered to the tenets of the Declaration of Helsinki.

### 2.1. Surgical Technique

All surgeries were performed under general anesthesia by a single experienced surgeon. A gauze soaked in 1:10,000 epinephrine was packed into the middle meatus to decongest the nasal mucosa. After upper punctum dilatation, a 23-gauge vitrectomy light pipe was inserted through the upper canaliculus into the lacrimal sac. Lidocaine with 1:100,000 epinephrine was injected into the nasal mucosa at the lacrimal fossa, where the lacrimal sac was located. The nasal mucosa was incised using an elevator and removed using ethmoid forceps. A Kerrison rongeur was used to remove the bone and expose the lacrimal sac. The lacrimal sac was tented using a light pipe and excised using a crescent blade. After removing the lacrimal sac using ethmoid forceps, lacrimal irrigation was performed to confirm the patency of the lacrimal passage. Bicanalicular silicone tube intubation was performed, and the anastomosis site was packed with biodegradable synthetic polyurethane foam Nasopore (Polyganics, Groningen, The Netherlands).

Postoperatively, all patients were prescribed eye drops (topical antibiotics and steroids) and budesonide nasal spray. Follow-up examinations were performed at 1 and 2 weeks and at 1, 2, 3, 4, and 6 months after surgery. The silicone tube was removed three months after surgery, and lacrimal irrigation was performed at each visit.

### 2.2. Main Outcome Measures

The types, durations, and total ingredient numbers of topical anti-glaucoma medications, along with the EDCR surgical outcomes, were reviewed in detail. If the topical anti-glaucoma medication ingredients were changed, the analysis was based on the ingredients used during the 6-month period immediately before surgery. To compare the effects of drug ingredients, we counted the ingredients included in the fixed-combination anti-glaucoma medications separately. When two ingredients, brimonidine and timolol, were mixed in one bottle, the number of drugs was counted as two. To quantify the impact of the long-term use of a single ingredient versus the short-term use of multiple drug ingredients, we multiplied the number of eye drop ingredients by the duration in months.

### 2.3. Statistical Analysis

Statistical analyses were performed using SPSS statistical software (version 23.0; IBM Corp., Armonk, NY, USA) and the R statistical package (version 3.5.0; R Foundation for Statistical Computing, Vienna, Austria). The independent t-test, chi-square test, and Fisher’s exact test were used to analyze baseline demographics. Univariate and multivariate logistic regression analyses were performed to evaluate the relationship between the ingredients of anti-glaucoma medications and the EDCR success rate and the risk factors associated with the EDCR success rate. Because of multicollinearity problem, stepwise backward elimination was performed to identify the independent factors. Fisher’s exact test was used to compare the EDCR success rate according to the number of ingredients, duration of glaucoma treatment, and the product of the number of ingredients multiplied by duration in months. An Edwards-Venn diagram was used to present patients’ numbers and success rates using topical anti-glaucoma medications based on their ingredients. The area under the receiver operating characteristic curve (AUROC) was calculated to compare the effects of several factors on the EDCR success rate. Statistical significance was set at *p* < 0.05.

## 3. Results

During the study period, EDCR was performed on 30 eyes of 30 patients receiving anti-glaucoma medication. A total of 120 patients were enrolled in this study, including 30 in the anti-glaucoma medication group and 90 age- and sex-matched controls. The demographic characteristics of the patients are shown in Table 1.

**Figure 1:**
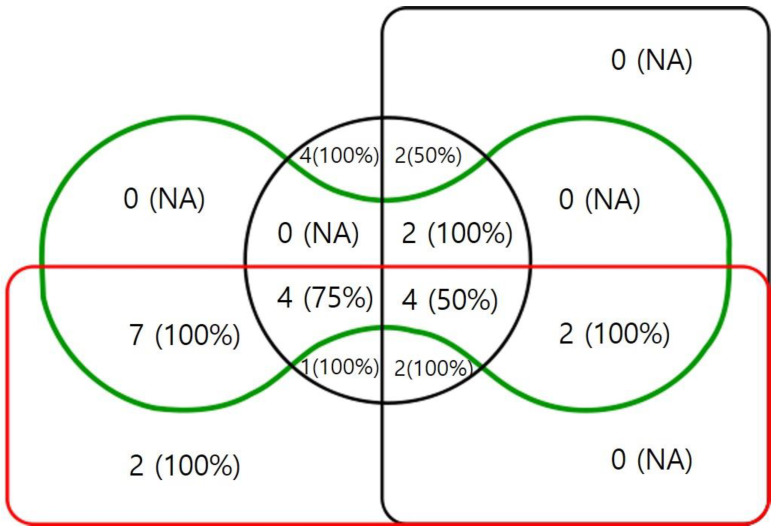
The Edwards-Venn diagram shows the number and the success rates (within parentheses) of patients using anti-glaucoma medication according to ingredients. Black box: alpha agonists. Red box: beta-blockers. Black circle: prostaglandin analogs. Green curve: carbonic anhydrase inhibitors. NA: not assessed.

The mean age was 68.1 years, and the female-to-male ratio was 3.3:1. No statistically significant differences were observed in age, sex, laterality, or underlying systemic diseases between the groups. In this study, patients were administered various combinations of anti-glaucoma ingredients. Consequently, we analyzed the number of patients and EDCR success rates of the four overlapping ingredients using an Edwards-Venn diagram (Figure 1). Six months after surgery, the success rates were 97.8% in the control group and 86.7% in the anti-glaucoma medication group (*p* = 0.034). When analyzed based on the glaucoma types, the success rates for the control group, normal tension glaucoma, primary open-angle glaucoma, pseudoexfoliation glaucoma (PXF), and chronic angle-closure glaucoma (CACG) were 97.8% (88/90), 90.0% (10/11), 85.7% (12/14), 100.0% (2/2), and 66.7% (2/3) (*p* = 0.035). However, the CACG group and the PXF group each consisted of only two patients. Also, in the post-hoc analysis, the CACG group did not exhibit statistical significance (*p* = 0.360). When analyzed based on the severity of glaucoma according to mean deviation in the visual field, the success rates for early glaucoma, moderate glaucoma, and severe glaucoma were 88.9% (8/9), 100.0% (8/8), and 76.9% (10/13) (*p* = 0.311).

Six months after surgery, the success rates were 97.8% (control group), 86.4% (beta-blockers), 75.0% (alpha-agonists), 84.2% (carbonic anhydrase inhibitors [CAI]), and 78.9% (prostaglandin [PG] analogs). Using univariate logistic regression analysis, alpha-agonists (odds ratio [OR], 0.086; 95% confidence interval [CI], 0.015–0.488, *p* = 0.006), CAI (OR, 0.163; 95% CI, 0.030–0.881, *p* = 0.035), and PG (OR, 0.076; 95% CI, 0.013–0.450, *p* = 0.005) were found to be negative risk factors for EDCR success rate (Table 2). Multivariate backward elimination was performed to analyze the ingredients that had the greatest impact on the EDCR success rate among the four ingredients of the anti-glaucoma medication. Multivariate logistic regression analysis revealed that PGs were the most influential risk factor for EDCR success among the anti-glaucoma medication ingredients (OR, 0.076; 95% CI, 0.013–0.450; *p* = 0.005). When analyzed according to subtypes of PG, the success rates for the control group, latanoprost, tafluprost, bimatoprost, and travoprost were 97.8% (88/90), 70.0 (7/10), 100.0% (5/5), 100.0% (2/2), and 50.0% (1/2) (*p* = 0.005). The success rates decreased in the latanoprost and travoprost group. However, in the post-hoc analysis, neither group exhibited statistical significance (*p* = 0.999 and *p* = 0.580), respectively.

We also analyzed the success rates according to the number of anti-glaucoma medication ingredients. Success rates were compared when one, two, three, and all four drug ingredients were used. The success rates were 97.8% (control), 100.0% (one ingredient), 90.0% (two ingredients), 90.0% (three ingredients), and 50.0% (all four ingredients) (*p* = 0.010). The success rate of patients, including all four anti-glaucoma medication ingredients, was also statistically significant in the post-hoc analysis (*p* = 0.008) (Table 3).

The success rates were also compared according to the duration of anti-glaucoma medication use. The success rates were 97.8% (control group), 91.7% (duration < 12 months), 100.0% (12–24 months), and 76.9% (>24 months) (*p* = 0.019). In the group of patients who used anti-glaucoma medication for >24 months, the success rate was significantly different among the three groups (*p* = 0.019). The post-hoc analysis yielded a *p*-value of 0.083, slightly higher than the significance level of 0.05 (Table 4).

When the number of drug ingredients was multiplied by the duration in months, the median value was 69. The anti-glaucoma medication group was divided into two groups based on median values. The success rates were 97.8% (control group), 93.3% (group 1, ≤69), and 80.0% (Group 2, >69). The EDCR success rate in Group 2 was significantly decreased (*p* = 0.022). The post-hoc analysis yielded a *p*-value of 0.061, slightly higher than the significance level of 0.05 (Table 5).

Univariate logistic regression analysis showed that the number of anti-glaucoma medications (OR, 0.437; 95% CI, 0.247–0.772, *p* = 0.004), duration of anti-glaucoma medication (OR, 0.981; 95% CI, 0.964–0.997, *p* = 0.020), and the product of ingredient numbers multiplied by duration in months (OR, 0.991; 95% CI, 0.983–0.997, *p* = 0.009) were negative risk factors for EDCR success rate. Multivariate backward elimination was employed to analyze the risk factors that had the greatest influence on the EDCR success rate. Multivariate logistic regression analysis indicated that the number of anti-glaucoma medications was the only risk factor for EDCR success rate (OR, 0.437; 95% CI, 0.247–0.772; *p* = 0.004) (Table 6).

**Figure 2:**
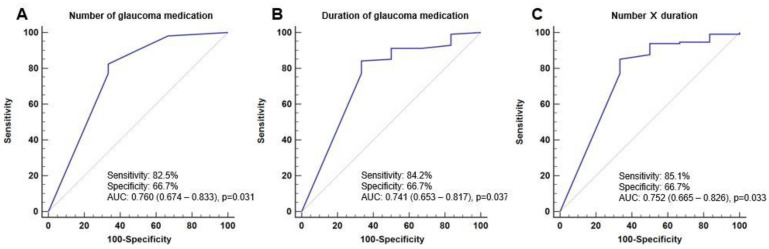
The ROC curves showing the success rates of endoscopic dacryocystorhinostomy accor–ing to risk factors. The ROC curves according to (**A**) number of glaucoma medication, (**B**) duration of glaucoma medication, and (**C**) the product of ingredient number multiplied by duration.

The receiver operating characteristic curve (ROC) curve of the number of anti-glaucoma medications showed a limited area under the ROC curve (AUC) of 0.760 (95% CI, 0.674–0.833, *p* = 0.031), with a sensitivity of 82.5% and a specificity of 66.7%. The ROC curves for the duration of anti-glaucoma medication use (AUC = 0.741; 95% CI, 0.653–0.817; *p* = 0.037) and the product of ingredient number multiplied by duration (AUC = 0.752; 95% CI, 0.665–0.826; *p* = 0.033) were similar. However, the AUC of the number of anti-glaucoma medications was higher than that of the other two variables (Figure 2).

## 4. Discussion

According to previous studies, 5–23% [2,3,4] of patients were receiving anti-glaucoma treatment when lacrimal drainage system obstruction was being diagnosed. In our study of 795 patients with NLDO, 30 (3.8%) used topical anti-glaucoma medications during EDCR surgery. Some studies have reported that anti-glaucoma medications induce inflammatory and fibrotic changes on the conjunctival surface. Likewise, they may induce inflammation and fibrosis in the epithelium and subepithelial tissue of the lacrimal drainage system, causing NLDO [4,13,14]. Furthermore, previous studies have reported that topical anti-glaucoma medications may exaggerate the scarring response in the mucosa of the lacrimal drainage system and affect the success rate of EDCR [3,9]. Di Maria et al. [9] hypothesized that the toxicity of anti-glaucoma medications induces fibrosis in the mucous membranes of the lacrimal system. Mandel et al. [3] demonstrated that nasal endoscopy in failed DCR surgery in patients treated with anti-glaucoma medications showed mucosal scarring at the anastomosis site. Our results also aligned with these hypotheses, as the success rate was low in the anti-glaucoma medication group (*p* = 0.034). We also thought there might be a difference in EDCR success rates depending on the glaucoma types or severity, but no significant difference was found. Although the success rate was relatively low in the CACG group (66.7%) and patients with severe visual field defects (76.9%), there was no statistical significance. Because the number of subjects in each group is small, detailed analysis is challenging, so a large-scale study is needed.

We aimed to exclude patients with severe deformities in the osteomeatal complex that could potentially decrease the success rate of DCR and ensure a representative sample of NLDO by obtaining orbital CT scans. The preoperative imaging helps in recognizing the bony anatomy surrounding the lacrimal outflow system, the mucous membranes, normal anatomic variants, sinusitis, tumors, and previous trauma [15]. The correlation between nasal septal deviation, sinusitis, and structural abnormalities of the sinonasal cavity and NLDO has been studied, but the results are still inconclusive [16]. Therefore, we did not exclude all minor anatomic variants, but we tried to exclude severe scar formation or synechia resulting from trauma, tumors, or previous operations, as they could potentially contribute to the failure of DCR.

Several studies have reported that certain ingredients of topical anti-glaucoma medications might have a greater effect on epiphora; however, a unified opinion has not been established. Although Oritz-Basso et al. [13] showed no evidence that any particular ingredient of an anti-glaucoma medication carries a higher risk of NLDO, other studies have indicated that specific ingredients of anti-glaucoma medications carry a higher risk. The ingredients were diverse, including timolol [4], dorzolamide [14], CAI [3], and PGs [9]; however, their hypothesis consistently suggested that anti-glaucoma medication ingredients influence the distal excretory lacrimal mucosa, leading to fibrosis. However, the precise mechanisms underlying inflammation and fibrosis remain unclear. In our study, alpha agonists, CAIs, and PGs were negative risk factors for the EDCR success rate. And PGs were the most influential risk factor for the EDCR success rate among glaucoma medication ingredients. Our results support the relationship between the use of topical PGs and fibrosis in NLD. Unlike other glaucoma medications, PG has generally been associated with intraocular inflammation because it can potentially induce further inflammatory responses. Therefore, the use of PG is recommended with caution in patients with an inflammatory history such as uveitis, herpes keratitis, and inflammatory glaucoma [17]. PGs likely trigger chronic inflammation by regulating immune cells and crosstalking with cytokines [18]. Endogenous PGs have a role in inflammatory mediation in the eyes and are considered a potent proinflammatory agent responsible for uveitis or cystoid macular edema [19]. The exact cause-and-effect relationship between PG-inducing exacerbations of uveitis or CME has not been elucidated. However, in animal studies, ocular inflammation was induced when a large amount of PG was administered to rabbit eyes [20], and there are numerous case reports supporting this. It can be inferred that these inflammatory mechanisms can also affect the NLD mucosa. Therefore, given the observed decreased success rate of EDCR in the PG group within this study, surgeons might contemplate switching preoperative anti-glaucoma medications to non-PG medications for concurrent glaucoma patients requiring DCR surgery.

We also hypothesized that the subtypes of PG might have different effects on the eyes. We analyzed the success rate of PG subtypes by classifying them into latanoprost, tafluprost, bimatoprost, and travoprost. However, due to the small patient numbers in each group, significant effects were not found. The adverse effects of PG, such as eyelid pigmentation, iris pigmentation, hypertrichosis around the eye, and deepening of the upper eyelid sulcus, are referred to as prostaglandin-associated periorbitopathy (PAP). Also, PAP was reported to be more frequent and severe in the bimatoprost group compared with other PG subtypes [21,22]. As reported by pharmacological studies, bimatoprost accumulates in higher concentrations in eyelid tissues than in the aqueous humor, iris, and ciliary body [23]. Therefore, bimatoprost might have the most negative impact on the eyelid compared with other PG subtypes. In contrast, an in vitro study revealed that bimatoprost exhibited lower cytotoxicity in conjunctiva-derived epithelial cells when compared with other PG subtypes [24]. Guenon et al. [24] reported that in commercial preparations, bimatoprost contains less benzalkonium chloride (BAK) than travoprost and latanoprost. Since ocular surface toxicity was associated with the concentration of the preservative BAK, bimatoprost showed lower cytotoxicity. Bimatoprost induced more side effects in the eyelid compared with other PGs; however, it exhibited lower toxicity in the conjunctiva. It is not known what side effects PG subtypes may induce in the NLD, and additional research is needed.

In addition to active ingredients, the preservatives in anti-glaucoma medications are also an issue. Topical anti-glaucoma medications are composed of active ingredients, preservatives, and excipients and are known to be associated with inflammatory and fibrotic changes on the ocular surface [5,6,7,8]. In particular, preservatives in anti-glaucoma medications destabilize bacterial cell membranes and have the same effect on normal corneal and conjunctival cells, thereby causing OSD [8]. OSD occurred more frequently in patients with increased glaucoma severity and was associated with higher exposure to BAK. A daily dose of BAK of more than three drops was an independent predictor of the OSD index score [25]. Although they did not directly compare preservative-containing and preservative-free glaucoma medications, several studies have suggested that active ingredients or preservatives in anti-glaucoma medications may contribute to the development of NLDO, similar to their role in inducing OSD [9,13]. However, Mandel et al. [3] reported no significant difference in the success rate of primary DCR between patients treated with preserved and those treated with non-preserved anti-glaucoma medications. In our study, all patients with glaucoma used preservative-containing eye drops. Therefore, assessing the impact of preservatives or excipients was impossible. However, it might be more advantageous to analyze the effects of active ingredients on NLD. Further studies are needed to investigate the potential effects of preservatives and excipients on the development of NLDO and the success rates of DCR. In our study, as it appeared that PG showed significant relevance to the success rate of DCR, it is necessary to compare the success rates of DCR among the control group, the non-preserved PG group, and the preserved PG group. Furthermore, when patients receiving anti-glaucoma treatment require DCR surgery, surgeons should consider switching from preservative-containing anti-glaucoma medications to preservative-free anti-glaucoma medications.

The cumulative dosage should also be considered, as glaucoma requires continuous eye-drop management. Variables such as the number of drugs, duration of use, and their combinations may contribute to cumulative effects. This study examined the success rate of EDCR regarding the number, duration, and cumulative effects of anti-glaucoma medications.

Seider et al. [4] reported that the number of topical anti-glaucoma medications was higher in the NLDO group than in the control group (1.58 vs. 0.73, *p* = 0.002). However, Ortiz-Basso et al. [13] reported that the proportion of patients using more than two anti-glaucoma medications was not higher in the NLDO group (67% vs. 62%, *p* = 0.491). In our study, patients treated with all four anti-glaucoma ingredients exhibited a decreased EDCR success rate (*p* = 0.008). However, the group of patients using all four anti-glaucoma ingredients consisted of only four patients. Therefore, the small number of patients may have introduced a bias. In the logistic regression analysis, the number of anti-glaucoma medications was the most relevant risk factor among the variables. We assumed the number of anti-glaucoma medications had the most prominent cumulative effect. In our study, we observed a reduced success rate when PGs were used. However, because PGs are the most commonly used first-line treatment, they are frequently part of the regimen for patients using multiple anti-glaucoma medications. Therefore, it is challenging to determine whether the reduced success rate is due to the presence of PGs or the cumulative effects of multiple eye drop ingredients.

The occurrence of NLDO after using anti-glaucoma medications has been mentioned as an idiosyncratic reaction in short-term use cases and a potential cumulative effect in long-term use cases; however, no statistically significant evidence has been provided [14]. In our study, patients using eye drops for >24 months showed a decreased EDCR success rate (*p* = 0.019). We assumed a cumulative effect of long-term use of anti-glaucoma medications.

According to our results, the number of ingredients and the duration of use contributed to a decrease in the EDCR surgical success rate. The authors observed a correlation between the number of ingredients and the duration of use, similar to the pack-year analysis in smokers. Consequently, we reanalyzed the product of these two factors as a single variable. The patients were divided into two groups based on the median value of the product obtained by multiplying the number of ingredients by the number of months of use. The success rate of EDCR was significantly decreased in Group 2 (>69) (*p* = 0.022). Although the group using anti-glaucoma medication for > 24 months and Group 2 (>69) were both statistically significant (*p* = 0.019 and *p* = 0.022, respectively), the post-hoc *p*-values were 0.083 and 0.061, respectively, which were slightly higher than the significant *p*-value of 0.05. However, it could be considered a borderline value in post-hoc analysis, indicating a potential tendency toward a decrease in the success rate of EDCR. When there are significant *p*-values implying overall differences among groups, post-hoc analysis is needed to identify which groups differ from each other. It is anticipated that with an increase in the number of patients, the *p*-values in post-hoc analysis might also become more evident. Although our study included more patients than previous studies, the number of patients with glaucoma was still insufficient for analyzing risk factors. Therefore, the small number of patients may have introduced bias. Considering that the post-hoc *p*-value was slightly higher than the significance level of 0.05, it may be necessary to confirm the significance by analyzing a larger number of patients.

We multiplied the number of drug ingredients by the duration in months to analyze the cumulative effects. AUROC was evaluated to compare which factor is more related to EDCR success rates. The ROC curves and AUC indicated similar relevance among the number of anti-glaucoma medication ingredients, duration of medication, and product of the number of drug ingredients multiplied by duration in months. The AUC of the number of anti-glaucoma medications was higher than that of the other two variables. Although the product of the number of drug ingredients multiplied by duration in months did not show better results than the other two variables in AUROC, this is the first study to analyze the cumulative effect of anti-glaucoma medications. Further discussion on appropriate methods for measuring cumulative effects is needed.

A limitation of our study is that all patients received topical anti-glaucoma medications containing preservatives, which could affect the success rate. Accurately excluding the effects of preservatives from the success rate of EDCR was challenging. In addition, the group using anti-glaucoma medications for more than 24 months and Group 2 (>69) were both statistically significant but not in the post-hoc analysis. The limitation of our study can be attributed to its relatively small sample size. Further prospective studies with larger sample sizes are required to confirm our findings.

In conclusion, the success rate of EDCR surgery decreased in patients receiving anti-glaucoma medications. PG-containing eye drops, a higher number of ingredients, a longer duration of use, and the cumulative effect of both the number and duration contributed to a decrease in the surgical success rate. A higher number of drug ingredients was the most influential factor among the variables. Therefore, when performing EDCR on patients using topical anti-glaucoma medications, surgeons should inform patients about the possibility of increased recurrence rates after EDCR surgery.

## Figures and Tables

**Table 1:** Demographics of the patients.

Characteristics	Anti-Glaucoma Medication Group (*n* = 30)	Control Group (*n* = 90)	*p*-Value
Age (years ± SD)	68.1 ± 9.9	68.1± 8.5	0.967 *
Gender (n, %)			
Male	7 (23.3)	21 (23.3)	0.999 †
Female	23 (76.7)	69 (76.7)	
DM	8 (26.7)	10 (11.1)	0.072 ‡
HTN	15 (50.0)	30 (33.3)	0.102 †
Laterality (right, %)	12 (40.0)	51 (56.7)	0.113 †

SD = standard deviation; DM = diabetic mellitus; HTN = hypertension. * Independent *t*-test, † Chi-square test, ‡ Fisher’s exact test.

**Table 2:** Risk factors affecting the success rate of EDCR based on anti-glaucoma medication ingredients.

	Univariate			Multivariate		
	B	OR (95% CI)	*p*-Value *	B	OR (95% CI)	*p*-Value *
Beta-blockers	−1.609	0.200 (0.037–1.067)	0.060			
Alpha agonists	−2.457	0.086 (0.015–0.488)	0.006			
Carbonic anhydrase inhibitors	−1.812	0.163 (0.030–0.881)	0.035			
Prostaglandin analogs	−2.580	0.076 (0.013–0.450)	0.005	−2.580	0.076 (0.013–0.450)	0.005

EDCR = endoscopic dacryocystorhinostomy; OR = odds ratio; CI = confidence interval; * Logistic regression analysis.

**Table 3:** Comparisons of EDCR success rates according to anti-glaucoma medication ingredients.

	Control Group (*n* = 90)	One Ingredient (*n* = 6)	Two Ingredients (*n* = 10)	Three Ingredients (*n* = 10)	Four Ingredients (*n* = 4)	*p*-Value
EDCR Success rates	88 (97.8%)	6 (100%)	9 (90.0%)	9 (90.0%)	2 (50.0%)	0.010 ‡
Post-hoc analysis		0.999 ‡	0.273 ‡	0.273 ‡	0.008 ‡	

EDCR = endoscopic dacryocystorhinostomy; ‡ Fisher’s exact test.

**Table 4:** Comparisons of EDCR success rates according to the duration of anti-glaucoma medication use in months.

	Control Group (*n* = 90)	≤12 Months (*n* = 12)	12–24 Months (*n* = 5)	>24 Months (*n* = 13)	*p*-Value
EDCR success rates	88 (97.8%)	11 (91.7)	5 (100.0)	10 (76.9)	0.019 ‡
Post-hoc analysis		0.316 ‡	0.999 ‡	0.083 ‡	

EDCR = endoscopic dacryocystorhinostomy; ‡ Fisher’s exact test.

**Table 5:** Comparisons of the success rates of EDCR based on the product of ingredient numbers multiplied by duration in months.

	Control Group (*n* = 90)	Group 1 (≤69) (*n* = 15)	Group 2 (>69) (*n* = 15)	*p*-Value
EDCR success rates	88 (97.8%)	14 (93.3)	12 (80.0)	0.022 ‡
Post-hoc analysis		0.746 ‡	0.061 ‡	

EDCR = endoscopic dacryocystorhinostomy; ‡ Fisher’s exact test.

**Table 6:** Univariate and multivariate logistic regression analyses for success rate of EDCR.

	Univariate			Multivariate		
	B	OR (95% CI)	*p*-Value *	B	OR (95% CI)	*p*-Value *
Age	−0.004	0.996 (0.907–1.094)	0.941			
Sex	−0.477	0.621 (0.108–3.577)	0.594			
DM	−0.132	0.876 (0.096–7.972)	0.907			
HTN	−0.539	0.583 (0.113–3.022)	0.521			
Mean deviation of VF	0.028	1.028 (0.911–1.160)	0.654			
Number of glaucoma medication	−0.829	0.437 (0.247–0.772)	0.004	−0.829	0.437 (0.247–0.772)	0.004
Duration of glaucoma medication	−0.020	0.981 (0.964–0.997)	0.020			
Product of ingredient numbers multiplied by duration in months	−0.010	0.991 (0.983–0.997)	0.009			

EDCR = endoscopic dacryocystorhinostomy; OR = odds ratio; DM = diabetic mellitus; HTN = hypertension; VF = visual field; * Logistic regression analysis.

## Data Availability

Data supporting the findings of the current study are available from the corresponding author on reasonable request.
